# The vicious cycle of dental fear: exploring the interplay between oral health, service utilization and dental fear

**DOI:** 10.1186/1472-6831-7-1

**Published:** 2007-01-14

**Authors:** Jason M Armfield, Judy F Stewart, A John Spencer

**Affiliations:** 1 Australian Research Centre for Population Oral Health, Dental School, Faculty of Health Sciences, The University of Adelaide, South Australia 5005, Australia

## Abstract

**Background:**

Based on the hypothesis that a vicious cycle of dental fear exists, whereby the consequences of fear tend to maintain that fear, the relationship between dental fear, self-reported oral health status and the use of dental services was explored.

**Methods:**

The study used a telephone interview survey with interviews predominantly conducted in 2002. A random sample of 6,112 Australian residents aged 16 years and over was selected from 13 strata across all States and Territories. Data were weighted across strata and by age and sex to obtain unbiased population estimates.

**Results:**

People with higher dental fear visited the dentist less often and indicated a longer expected time before visiting a dentist in the future. Higher dental fear was associated with greater perceived need for dental treatment, increased social impact of oral ill-health and worse self-rated oral health. Visiting patterns associated with higher dental fear were more likely to be symptom driven with dental visits more likely to be for a problem or for the relief of pain. All the relationships assumed by a vicious cycle of dental fear were significant. In all, 29.2% of people who were very afraid of going to the dentist had delayed dental visiting, poor oral health and symptom-driven treatment seeking compared to 11.6% of people with no dental fear.

**Conclusion:**

Results are consistent with a hypothesised vicious cycle of dental fear whereby people with high dental fear are more likely to delay treatment, leading to more extensive dental problems and symptomatic visiting patterns which feed back into the maintenance or exacerbation of existing dental fear.

## Background

Despite reductions in pain associated with dental visits and an increased awareness by dentists of the importance of building trusting relationships, dental fear remains a major issue for dental clinicians and their patients [1]. Dental fear has long-term implications because it is both reasonably stable and difficult to assuage [2]. The significance of dental fear as an issue in dentistry is magnified by the high prevalence of dental fear reported in many countries. Child dental fear has been reported to be as high as 43 per cent [3] in some countries while estimates of the prevalence of high dental fear among Australian adults are about 16 per cent [4,5]. Both the high prevalence of dental fear and the ramifications in terms of disease experience and treatment make it important that we better understand the mechanisms by which dental fear is maintained and possibly exacerbated.

A number of studies have found an association between dental fear and both visiting patterns and disease experience. For example, Schuller et al. [6] found that individuals with high fear visited the dentist less often and had more decayed and more missing teeth. Similarly, Thomson et al. [5] found associations between dental fear and less frequent dental visiting, increased visiting for a problem and increased social and functional impairment. Similar findings have been reported in other research [7,8].

**Figure 1:**
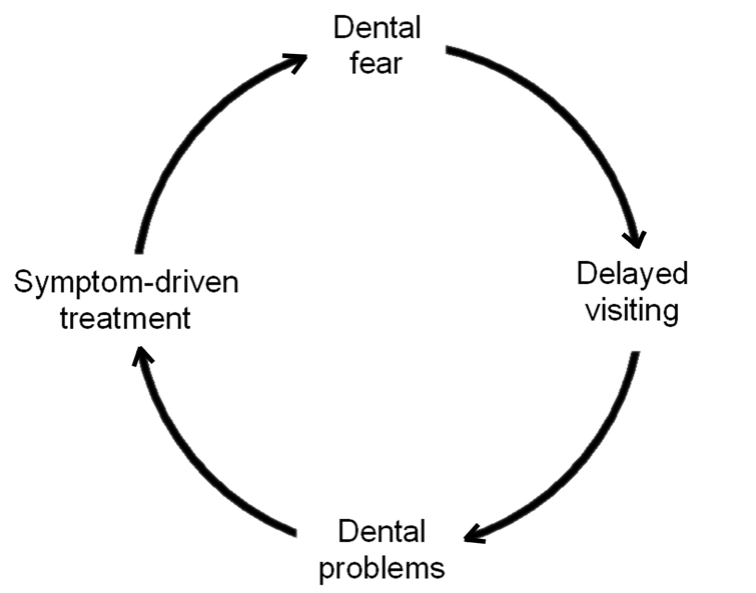
Model of the vicious cycle of dental fear.

The idea of a vicious cycle of dental fear has been promulgated by several studies [5,9-16]. Some researchers posit a role for psychological variables such as embarrassment, with dental fear and anxiety leading to avoidance, a deterioration in dental health, and feelings of shame and embarrassment culminating in reinforced avoidance [17,18]. In contrast, Bouma et al. [14] propose that anxiety plays a crucial role in avoidance behaviour by causing a deterioration in oral health and an increase in the perceived likelihood of pain and restorative treatments resulting in further negative dental visiting experiences. Similarly, Thomson et al. [5] have argued that dental fear may be a component in a cycle of dental disadvantage, with dentally anxious individuals avoiding dental care and thereby worsening their problems and increasing the likelihood that subsequent dental visits will be for emergency reasons. These conceptualisations share the common feature that the dental fear is believed to feed back into itself as a result of a number of repercussions of the fear (Figure 1). While it may be argued that being forced to seek help as a result of an acute dental problem, most likely due to toothache, provides an opportunity for an individual to confront their feared situation and therefore reduce their fear, given the likelihood of painful and invasive treatment associated with the visit it is likely that any positive benefit from exposure would be mitigated by the aversive treatment experiences.

References to the concept of a vicious cycle of fear are replete within the psychological literature, however no systematic effort has yet been made to apply this idea in an analytical fashion to dental fear. For the most part, the idea of a 'vicious cycle' has been used post hoc to explain the relationship between dental fear and dental visiting behaviours without any substantive effort to explore the chain of relationships presupposed by the concept. The aim of this study was, therefore, not only to explore, within a contemporary Australian population, the relationship between dental fear and dental visiting patterns, prevalence of dental problems and symptom-driven treatment but to examine the hypothesised sequence of the 'vicious cycle' of fear, whereby dental fear, delayed dental visiting, increased dental problems and symptom-driven treatment form a linked chain feeding back into the fear experience.

## Methods

The data for the study are derived from the 2002 National Dental Telephone Interview Survey (NDTIS) [19] that used computer assisted telephone interviews of a random sample of people across Australia aged five years and over. Telephone numbers were randomly sampled from an electronic 'white pages' listing and grouped into 13 separate samples or strata. The mainland state capitals of Sydney, Melbourne, Brisbane, Perth and Adelaide as well as rest-of-state for each of the capital cities respectively comprised 10 separate strata. The remaining strata consisted of the state of Tasmania and the two largest mainland territories, the Northern Territory and the Australian Capital Territory.

In an effort to reach unlisted telephone numbers, a random digit substitution was applied to the final digit of each telephone number sampled, as described by Frankel and Frankel [20]. Where possible, these numbers were back matched to the electronic white pages to obtain addresses. Telephone numbers were designated as 'unlisted' if they lacked electronic white pages phone listings and corresponding addresses while telephone numbers were deemed to be 'listed' if there was a matching phone number in the white pages directory. In the state capital and rest-of-state strata the target number of participants was 600, while the target numbers in the smaller jurisdictions of Tasmania, and each of the mainland territories were 450 and 400 respectively.

Survey methods were based on those recommended by Dillman [21]. A primary approach letter (PAL) was sent to the address accompanying each sampled telephone numbers about 10 days before initial telephone contact. The PAL contained information regarding the study and the anticipated time of telephone contact. Each sampled number was initially called up to a maximum of six times, after which the number was abandoned if there was no answer. To ensure that the household was in scope and to select a target person, a standard procedure was followed upon successfully contacting a household. First, telephone numbers belonging to anything other than a residential dwelling were excluded. Second, if only a single person resided at the residence they were selected for participation. Third, if more than one person resided at the dwelling, respondents were randomly selected based on them being either the person in the household having the next birthday or the person with the most recent birthday. When a target person was identified up to six more calls were made, if necessary, in an effort to contact that person.

Participants were asked a structured series of questions which were based on previous rounds of the NDTIS. Pilot testing of the questions and interview procedures was carried out on a random selection of households from the city of Adelaide in South Australia and any modifications based on this testing incorporated into the telephone interviewing procedure prior to formal data collection. Interviewers were trained and all interviews were conducted in the presence of a supervisor.

Participants aged 16 years or over were asked a sequence of questions that accorded to one of two schedules. Schedule 1 interviews were presented to people who agreed to participate in the study while Schedule 2 interviews were undertaken when a selected person was unable to answer for themselves (for example due to illness, temporary absence from the house or language barriers) and were answered by an adult proxy. Where no proxy existed for a Schedule 2 interview, and so as not to exclude people who had poor English language skills, a small number of interviews with selected participants were conducted in other languages.

The interview schedules contained a number of items relating primarily to use of dental services, treatment outcomes, insurance characteristics, and socio-demographic characteristics. The Australian Government Department of Health and Aged Care funded the NDTIS with the majority of the study questions addressing research on 'adult access to dental care'. Only a selection of the results from the full interview schedule is presented here.

To assess dental fear, participants were asked the single-item Dental Anxiety Question (DAQ) "Are you afraid of going to the dentist?". This relatively simple single-item measure of dental fear has previously been found to have good reliability and validity [22,23]. Single-item fear measures have been used in other epidemiological research and demonstrate good agreement with more commonly used multi-item dental fear scales [24]. The four response categories of the DAQ are 'Not at all', 'A little', 'Yes, quite' and 'Yes, very'.

Dental visiting characteristics were assessed via several items. Delays in visiting were measured by questions pertaining to previous service use: "How long ago did you see a dental professional about your teeth, dentures or gums?" and "How often on average would you seek care from a dental professional?". Intentions regarding future service use were measured using two additional questions: "When do you expect to make your next dental visit?" and "Do you have an appointment set for a check-up within the next 18 months?".

Dental problems were assessed using a number of approaches. First, subjects were asked to count either the number of missing or remaining teeth in their mouth. Second, people were asked if they felt they currently needed to have any fillings, any extractions, a scale and clean, a denture made or repaired, a check-up, gum treatment, or a dental crown or bridge. The studies participants were also asked a series of questions regarding the social impact of their dental condition. They were asked how often in the past 12 months they had had a toothache, felt uncomfortable about the appearance of their teeth, mouth or dentures, had to avoid eating some foods because of dental problems, felt that life in general was less satisfying because of dental problems or had had trouble sleeping because of dental problems. Responses were recorded on a 5-point scale ranging from 'Very often' to 'Never during the last 12 months' with an additional option of 'don't know'. Items were based on social impacts assessed by the Oral Health Impact Profile [25,26]. Finally, people were asked a global question regarding their oral health: "How would you rate your own dental health?".

Symptom-driven treatment seeking was assessed by questions relating to the reason for a person's most recent dental visit and the reason for their usual dental visiting. For those people who responded that their usual reason for visiting a dental professional was when they had a dental problem, they were asked the additional question "Would your dental visits usually be (necessary) for the relief of pain?".

The data obtained were weighted for two purposes: first, to account for differing sampling probabilities due to variations in both household size and state/territory population and second, to ensure that the sample for each stratum more accurately represented the population of the corresponding stratum, by also weighting by age and sex. The weights result in reported frequencies corrected for differences in probability of selection while maintaining the same sample size [19].

Data analysis comprised three steps. First, associations between dental fear and sociodemographic and dental characteristics, dental health and dental visiting patterns were examined. Second, the associations assumed by a vicious cycle of dental fear were analysed. Finally, a multivariate model was constructed to test the independent association of dental fear and other possible confounders with the vicious cycle profile.

The NDTIS was approved by the Australian Institute of Health and Welfare Ethics Committee. All participants were informed that they had the right to refuse to answer any question and that they would not be individually identifiable in regards to the results of the study.

## Results

A total of 24,938 unique telephone numbers were called in the NDTIS. A large proportion of the unlisted numbers were either out of service (*n *= 6,596), or out of scope predominantly due to being a business number (*n *= 3,923). Of the remaining 14,419 households deemed as in scope 3,141 resulted in non-contact after the six call attempts while participation was refused in a further 3,966 households. As a result of the random digit substitution, a total of 21.3% of participants were from an unlisted household. Overall, 7,312 participants providing completed interviews with a final participation rate of 64.8%. After excluding children aged 15 years old and younger, a final NDTIS study population of 6,112 people aged 16 years and over was obtained. The average age of this sample was 44.2 years (SD = 18.1, range = 16–98 years of age).

Table 1 presents a comparison of the sample characteristics with those of the Australian population as derived from the Australian census in 2001. There was good similarity between population characteristics and those of the sample.

**Table 1:** Comparison of NDTIS 2002 sample characteristics with population statistics derived for Australia from the 2001 national census

	**NDTIS 2002 (%)**	**Australia 2001 (%)**
Age*		
16–24 years	13.1	13.0
25–44 years	32.2	31.9
45–64 years	24.8	24.7
65+ years	13.4	13.6
**Male**	49.1	49.2
Household income < $20,000 per year**	20.3	21.2
Employed***	58.8	56.6
**Speaks English at home**	87.6	84.0
**Born in Australian**	76.0	76.8

* Percentages based on total population aged 5 years +

** Australia 2001 figure refers to household income < $400 per week which translates to < $20,800 per year

*** Australia 2001 figure refers to persons aged 15 years and over

In response to the single-item DAQ, 67.7% of participants responded 'Not at all', 15.1% responded 'A little', 5.2% said 'Yes, quite', and 11.9% stated 'Yes, very'. A number of socio-demographic differences were observed between dental fear groups (Table 2). Higher dental fear was associated with being dentate, female, having part-time employment or being unemployed, and having an annual household income of between $20,000 and $50,000 per year. Dental fear was also associated with age, increasing across age groups up to 46–64-year-olds but then decreasing among those aged 65+ years old. The association between fear and country of birth and speaking a language other than English at home was not statistically significant. Similarly, residential remoteness as measured by the Accessibility/Remoteness Index of Australia (ARIA) [27] and based on road distance to service centers was not significantly related to dental fear.

**Table 2:** Socio-demographic and dental characteristics by dental fear

	**Not afraid**		**A little afraid**		**Quite afraid**		**Very afraid**	
				
	*n*	%	*n*	%	*n*	%	*n*	%
Dentate status***								
Dentate	3,482	90.3	826	96.2	295	99.0	648	95.3
Edentulous	375	9.7	33	3.8	3	1.0	32	4.7
Sex***								
Male	2,024	52.4	332	38.6	124	41.6	218	32.1
Female	1,837	47.6	529	61.4	174	58.4	462	67.9
Age***								
16–24 years	633	16.4	160	18.6	45	15.2	61	9.0
25–39 years	1,117	28.9	269	31.2	90	30.3	191	28.1
40–64 years	1,417	36.7	341	39.6	135	45.5	349	51.3
65+ years	695	18.0	92	10.7	27	9.1	79	11.6
**Country of birth**								
Australia	2,958	76.6	647	75.1	231	77.5	514	75.6
Other	903	23.4	214	24.9	67	22.5	165	24.3
**Language spoken at home**								
LOTE	461	11.9	121	14.1	33	11.1	75	11.0
English	3,401	88.1	740	85.9	265	88.9	604	89.0
Employment*								
Full-time	1,577	42.9	365	44.4	135	47.4	261	39.0
Part-time	673	18.3	148	18.0	64	22.5	145	21.6
Not employed	1,422	38.7	309	37.6	86	30.2	264	39.3
Annual household income**								
Less than $20,000	824	24.3	151	19.8	52	20.2	141	23.5
$20,001 – $50,000	1,111	32.8	236	30.9	97	37.7	239	39.8
$50,001 – $80,000	783	23.1	210	27.5	62	24.1	123	20.5
Greater than $80,000	672	19.8	166	21.8	46	17.9	97	16.2
**Residential remoteness**								
Highly accessible	2,586	67.2	590	68.7	204	69.2	455	67.5
Accessible	799	20.8	179	20.8	66	22.4	151	22.4
Moderately accessible	401	10.4	83	9.7	22	7.5	55	8.2
Remote	50	1.3	5	0.6	3	1.0	8	1.2
Very remote	10	0.3	2	0.2	0	0.0	5	0.7

* χ^2 ^< 0.05, ** χ^2 ^< 0.01, *** χ^2 ^< 0.001

Note: Dentate refers to at least one natural tooth present in the mouth

Dental fear was associated with having had a longer time since the last dental visit and a greater average time between visits (Table 3). While 56.5% of people with no dental fear last visited the dentist in the previous 12 months, 46.2% of people who were very afraid of visiting the dentist reported last visiting within the previous year. Looking at average visiting frequency, 44.1% of people who rated themselves as very afraid visited the dentist less than once every two years on average compared to approximately 30% of individuals with no dental fear. In terms of future visiting patterns a similar trend was observed, with 76.9% of people who were very afraid expecting to make a dental visit in the next year, compared to 66.7% of people with no dental fear. In relation to when people expected to make their next dental visit, perhaps the most striking difference was that 27.6% of people who were very afraid of the dentist expected to make their next visit only when they experienced pain or a problem, compared to less than 17% of people with less dental fear. Almost 17% of the no dental fear group had an existing appointment to see a dentist compared to only 11.4% of the very afraid group.

**Table 3:** Dental visiting characteristics by dental fear

	**Not afraid**		**A little afraid**		**Quite afraid**		**Very afraid**	
				
	*n*	%	*n*	%	*n*	%	*n*	%
Time since last dental visit**								
Less than 12 months	2,169	56.5	456	53.3	176	59.1	311	46.2
1 year to < 2 years	682	17.8	186	21.7	54	18.1	134	19.9
2 years to < 5 years	505	13.2	113	13.2	46	15.4	112	16.6
5 years to < 10 years	237	6.2	52	6.1	12	4.0	53	7.9
> 10 years ago	246	6.4	49	5.7	10	3.4	63	9.4
Average visiting frequency**								
More than twice a year	976	26.6	189	22.7	82	29.4	107	17.2
Once per year	1,051	28.6	252	30.3	66	23.7	132	21.2
Once every 2 years	559	15.2	168	20.2	52	18.6	109	17.5
> Once every 2 years	1,083	29.5	224	26.9	79	28.3	274	44.1
When expected to make next visit**								
Less than 6 months	2,182	58.7	511	61.2	181	61.6	348	54.0
6 months to < 12 months	678	18.2	171	20.5	57	19.4	82	12.7
1 years to < 2 years	129	3.5	24	2.9	6	2.0	24	3.7
2 years to < 5 years	69	1.9	8	1.0	5	1.7	8	1.2
Greater than 5 years	35	1.0	3	0.3	4	1.3	4	0.7
Only for pain or a problem	626	16.8	118	14.1	41	13.9	178	27.6
Has an appointment for a future dental visit*								
Yes	664	17.2	129	15.0	55	18.5	77	11.4
No	3,191	82.8	731	85.0	243	81.5	598	88.6

* χ^2 ^< 0.05, ** χ^2 ^< 0.01, *** χ^2 ^< 0.001

Note: Dentate refers to at least one natural tooth present in the mouth

People who were very afraid of visiting the dentist had significantly more teeth missing than did people with less extreme dental fear (Table 4). Confining analyses to the maxillary arch, people with the most dental fear had significantly more missing teeth than people with either no dental fear or a little dental fear. Similarly, those people who were very afraid of going to the dentist had significantly more missing teeth in the mandibular arch than those people who were not afraid, were a little afraid or were quite afraid of going to the dentist.

**Table 4:** Mean number of teeth missing due to dental caries by dental fear

	**Maxillary arch**		**Mandibular arch**	
		
**Afraid of the dentist**	Mean	SD	Mean	SD
**Not at all**	2.73^a^	4.02	2.11^a^	2.78
**A little**	2.60^b^	3.68	1.94^b^	2.39
**Yes, quite**	3.01	4.15	1.89^c^	2.39
**Yes, very**	3.61^a,b^	4.66	2.61^a,b,c^	2.78
	*F *= 9.76, *p *< 0.001		*F *= 9.01, *p *< 0.001	

Note: Superscripts indicates significant Scheffe post-hoc differences, *p *< 0.05.

**Figure 2:**
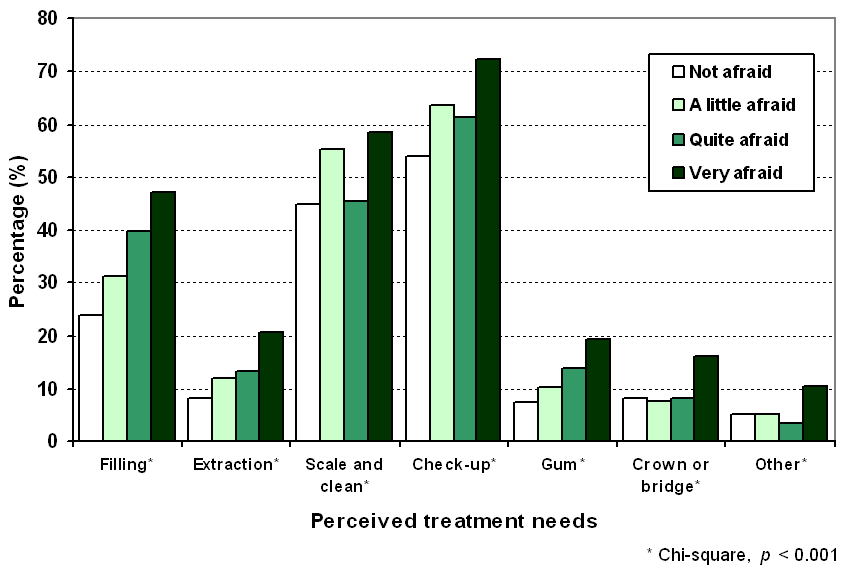
Perceived treatment needs by dental fear.

Higher self-rated dental fear was associated with significantly greater perceived need for fillings, tooth extraction, a scale and clean, a check-up, gum treatment, a dental crown or bridge and other treatment (Figure 2). There was a linear relationship between dental fear and perceived need for a filling, an extraction and gum treatment.

**Figure 3:**
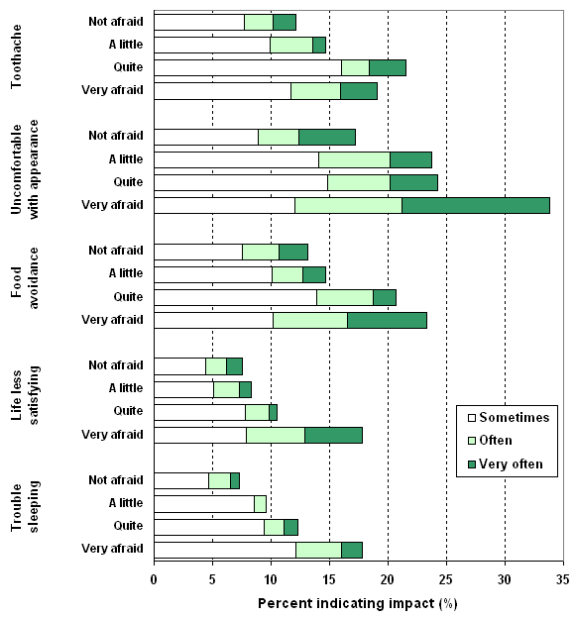
Psychosocial impacts of problems with teeth, mouth, or dentures during the previous 12 months by dental fear.

The distribution of responses to questions assessing the social impact of problems with the teeth, mouth or dentures of people with different levels of dental fear are shown in Figure 3. Dental fear was associated with a higher prevalence of toothache (χ^2 ^= 64.35, *p *= 0.001), more discomfort with the appearance of teeth, mouth or dentures (χ^2 ^= 184.16, *p *< 0.001), more frequent food avoidance due to dental problems (χ^2 ^= 108.11, *p *< 0.001), finding life less satisfying because of dental problems (χ^2 ^= 127.12, *p *< 0.001) and more trouble sleeping as a result of dental problems (χ^2 ^= 78.15, *p *< 0.001). Not only did people with very high dental fear report these impacts more often than did people with lower fear, but the ratings were more extreme with more people with very high fear stating that these social impacts occurred 'very often' than did people with less or no dental fear.

**Figure 4:**
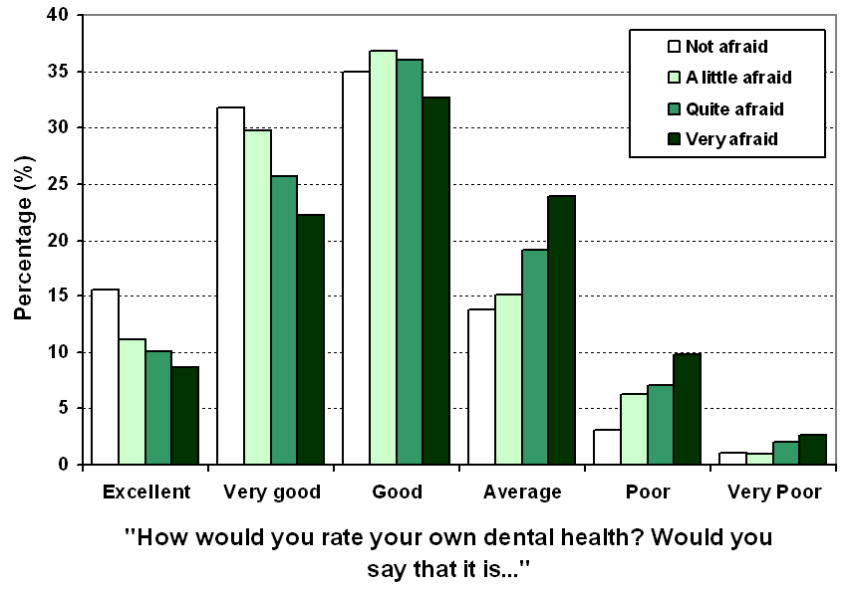
Self-rating of dental health by dental fear.

Participants made a global assessment of their oral health in response to the question "How would you rate your own dental health?" Just over 45% of people with no dental fear rated their dental health as being excellent or very good compared to 30.9% of people who were very afraid of going to the dentist (Figure 4). Conversely, people with the most dental fear were more likely to rate their dental health as average, poor or very poor (36.4%) in contrast to people who were not afraid, a little afraid or quite afraid (17.7%, 22.2% and 28.3% respectively). The association between dental fear and self-rated oral health was statistically significant, χ^2 ^= 178.95, *p *< 0.001

Some 61.3% of people who were very afraid of going to the dentist reported that the reason for their most recent visit in the last 12 months was for a problem, compared to 47.2% of people with no fear, 53.5% of people with a little fear and 59.4% of people who were quite afraid of going to the dentist (χ^2 ^= 31.09, *p *< 0.001). In addition, 67.3% of people with very high dental fear reported that their usual reason for a dental visit was for a problem compared with only 44.9% of people with no dental fear, 47.1% with a little fear and 45.8% who were quite afraid (χ^2 ^= 121.03, *p *< 0.001). Of those people who usually visited the dentist for a problem 72.3% of people who were very afraid of going to the dentist stated that the problem was usually for the relief of pain, compared to 54.7% of people with no dental fear, 67.2% with a little fear and 61.7% who were quite afraid (χ^2 ^= 57.72, *p *< 0.001).

Given that dental fear showed a relationship with delayed visiting patterns, poorer dental health and symptom-driven treatment, justification was provided for examining the cyclical process that is proposed as characterising the maintenance of dental fear. Specifically, we examined the relationship between fear and delayed visiting, the relationship between delayed visiting and dental problems, the relationship between dental problems and symptom-driven treatment, and finally the relationship between symptom-driven treatment and fear. Complete information on delayed visiting, dental problems, and usual reason for visiting was available for the 3,615 non afraid, 826 a little afraid, 271 quite afraid and 612 very afraid individuals.

People with high dental fear were significantly more likely to have a delayed visiting pattern, with a significantly higher percentage last visiting a dentist at intervals of greater than 2 years (43.9%) in comparison to people who were not, a little or quite afraid (29.1%, 26.5% and 27.7% respectively), χ^2 ^= 62.65, *p *< 0.001. In turn, those people with a longer time since their last dental visit had significantly more dental problems. People who last visited the dentist more than two years previously were significantly (Chi-square tests, *p *< 0.001) more likely to perceive themselves as needing a filling (39.4%), an extraction (18.6%) or gum treatment (12.6%) in contrast to people who had last visited within 2 years (23.7%, 7.3%, and 7.9% respectively). Of those people who perceived themselves as in need of dental treatment, determined here by anybody who responded that they needed either a filling, an extraction or gum treatment, 61.1% usually visited the dentist for an emergency treatment in comparison to 36.8% of people without a perceived need for a filling, extraction or gum treatment, χ^2 ^= 330.58, *p *< 0.001. Finally, a significantly greater percentage of people who usually visit the dentist for an emergency were very afraid of going to the dentist (16.3%), compared to people who normally visit for a check-up (7.3%), χ^2 ^= 106.02, *p *< 0.001.

**Figure 5:**
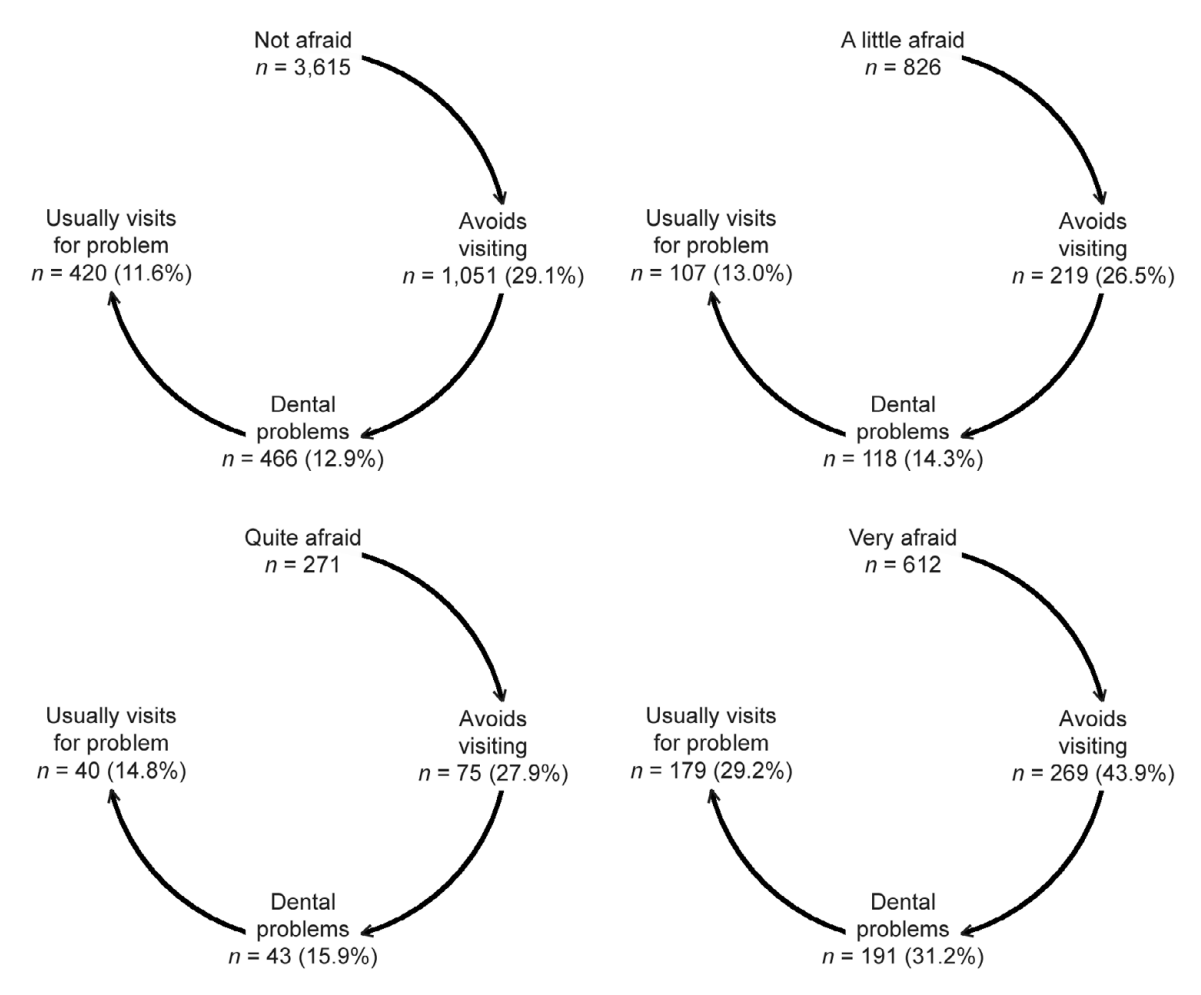
Following the path of the vicious cycle by categories of dental fear.

A graphical presentation of the concept of a vicious cycle for the four fear groups is shown in Figure 5. The figure shows the number and percentage of people in each fear group, after each component of the vicious cycle, who still fit the profile at that given point in the cycle. Overall, 179 people or 29.2% of those who were very afraid of going to the dentist fitted the profile of having delayed dental visiting, dental problems, and symptom-driven treatment seeking. This can be contrasted to the 11.6% of the group with no dental fear who exhibited the same characteristics.

Because dental fear was shown to vary by individual characteristics (see Table 2), a multivariate logistic regression was carried out to see if the difference between dental fear groups in fitting a vicious cycle profile was accounted for merely by differences in socio-demographic and dentate status variables between groups with differing levels of dental fear. The odds ratio of very fearful individuals having delayed dental visiting, dental problems, and symptom-driven treatment seeking was 3.33 (95% confidence interval 2.67–4.15) that for people without dental fear (Table 5). This effect for dental fear was statistically significant even though sex, dentate status, employment status and annual household income exhibited significant associations with fitting a profile consistent with being part of a vicious cycle. The odds ratios for people who were a little afraid or quite afraid of going to the dentist (ORs = 1.24 and 1.40 respectively) were in the expected direction but not statistically significant.

**Table 5:** Logistic regression model of characteristics associated with a vicious cycle profile (having delayed dental visiting, dental problems and symptom-driven treatment)

	**Odds ratio**	**Confidence interval**	**P-value**
**Fear of going to the dentist**			
Not afraid (Ref.)	1		
A little afraid	1.24	0.97–1.59	0.087
Quite afraid	1.40	0.96–2.04	0.079
Very afraid	3.33	2.67–4.15	<0.001
**Sex**			
Female (Ref.)	1		
Male	1.96	1.63–2.36	<0.001
**Age**			
16–24 (Ref.)	1		
25–39	1.28	0.95–1.73	0.100
40–64	0.99	0.74–1.32	0.918
65+	1.10	0.75–1.61	0.642
**Dentate status**			
Edentulous (Ref.)	1		
Dentate	1.71	1.25–2.35	0.001
**Employment**			
Student/retired (Ref.)	1		
Unemployed	1.49	1.09–2.03	0.013
Part-time	1.48	1.05–2.07	0.023
Full-time	1.62	1.18–2.22	0.003
**Annual household income**			
Less than $20,000 (Ref.)	1		
$20,001 – $50,000	0.64	0.50–0.82	<0.001
$50,001 – $80,000	0.48	0.36–0.64	<0.001
Greater than $80,000	0.32	0.23–0.45	<0.001

## Discussion

It was proposed that a vicious cycle exists in relation to dental fear whereby the behavioural and symptomatic consequences of dental fear ultimately lead to its maintenance and possible exacerbation. While causality can obviously not be established in a study such as this, the results of the current study are consistent with the notion of a vicious cycle of dental fear whereby the delaying of dental visits is related to increased dental problems which is related to increased invasive emergency treatment which, in turn, is related to greater dental fear and anxiety. A significant association was shown for each link in the proposed vicious cycle.

Dental fear was found to be related to less frequent dental visiting, whether measured by past behaviour or future intentions, more prevalent dental problems, whether assessed by the number of teeth missing, perceived need, social impact or self-rated oral health, and symptom-driven treatment as measured by a person's usual reason for visiting. These findings support those of a number of other studies both within Australia and elsewhere. In Australia, for example, Thomson et al. found higher dental fear for people who last visited the dentist more than 2 years ago, who usually visited for a problem, and who experienced social impacts resulting from their oral health state [5].

People who were very afraid of going to the dentist had more missing teeth than did people with less or no fear. The number of missing teeth has previously been found to be a more sensitive marker of dental fear than the traditional measure of the number of decayed, missing or filled teeth (DMFT). For instance, Schuller et al. found that while there was no statistically significant difference between the DMFT scores of individuals with high or low dental fear, the number of missing teeth was almost 50% higher among high dental fear than among low dental fear people [6]. This was interpreted as a preference for high fear people to have their teeth extracted instead of restored. However, it is also possible that the increased number of teeth extracted might be as a result of differences in the progression of carious lesions between people with high and low fear when they finally seek treatment. This fits well with the belief of Bouma et al. that if the vicious cycle of fear, treatment need and negative treatment experience is not broken the eventual consequence is full mouth extraction [14].

In many countries, use of dental services may be strongly related to access to oral health care. In Australia, at least theoretically, dental services are universally available. Publicly funded dental care, however, is rationed and available to those earning less than a specified income, who are on an invalid or old-age pension or who are war veterans. About one-quarter of Australians are eligible to received public-funded dental care [19]. Income strongly affects access to services in relation to both private dental services and public dental care which may be characterised by lengthy waiting lists. It is therefore not surprising that this study found household income to be significantly associated with the vicious cycle phenomenon. People on lower income invariably have both increased oral disease experience and more barriers to accessing dental care. Nonetheless, even after controlling for household income, dental fear was significantly associated with having characteristics associated with a vicious cycle.

Numerous studies support the idea that dental fear can result from previously traumatic or negative dental experience [28-31]. The subsequent association of dental visiting with aversive consequences is an example of classical conditioning learning. However, cognitive factors are suggested by findings that many highly anxious people can not recall an aversive event which might explain the origin of their dental anxiety [32]. In relation to other fears, the perception of vulnerability associated with the feared object is seen as critical in the determination of fear [33]. Avoidance of dental visits not only leads to the potential progression of caries or periodontal disease but also prevents people from 'extinguishing' the anxious or fearful state as a result of non-traumatic dental experiences.

One of the major limitations of this study is its cross-sectional design, which means that causality cannot be inferred from the results. While the results provide support for the existence of a vicious cycle in relation to dental fear, no information was available on the temporal sequencing of events. In order to test for causality a longitudinal study design is required, and this is currently being planned by the authors. However, interpretation of results from this study should be tempered by the realisation that more research is required before the causality implied by the term 'vicious cycle' is confirmed.

Interestingly, 11.6% of people with no dental fear also fitted the profile described by a vicious cycle of dental fear. It is likely that these people fall into the avoidance-problem-symptomatic visiting pattern as a result of different set of reasons. Research has shown that many people delay dental visiting due to issues of cost [19,34], perceived time restraints [35] or out of apathy or lack of interest [36,37]. These factors may have affected service utilization by low-fear people in the current study, with implications similar to those of high-fear people who have infrequent dental service utilization. This study found that 40% of people with no dental fear who had delayed dental visiting patterns also had a perceived dental problem and usually visited for a problem. Not visiting a dentist is related to having dental problems regardless of dental fear, although a difference in negative outcome is evident across dental fear groups.

Another interesting finding was that almost 70% of people who described themselves as being very afraid of going to the dentist did not have delayed dental visiting, a perceived problem and symptomatic visiting patterns. It is certainly not the case that having high dental fear is a necessary and sufficient precondition for poor oral health outcomes. Indeed, the percentage of very fearful individuals fitting a vicious cycle profile would most likely be much higher if people with dental phobias rather than just dental fears were examined. The Diagnostic and Statistical Manual of Mental Disorders [38] distinguishes phobia from fear on the basis of the feared stimulus being avoided, or else endured with intense distress, and that the fear or avoidance results in significant impairment or distress. Given that about two-thirds of all people indicating a high dental fear also reported visiting the dentist on average at least once every two years, there is good reason to believe that although these people might have reported being very afraid of going to the dentist the majority of them would not be classified as being dentally phobic. Indeed, it is precisely those people who reported high dental fear, avoidance of visiting the dentist and significant social and functional impacts, who appear to meet the criteria for dental phobia.

Another factor that may play a central role in differentiating very dentally fearful people who fit the vicious cycle profile from those who do not is differential use of coping strategies. However, and despite a well-developed literature on coping in relation to many pain and anxiety disorders, very little work has looked at coping strategies and dental fear. In regards to children, it appears that the level of dental fear and the experience with pain at the dentist is significantly associated with both ability to cope and with choice of coping strategies [39]. Distraction is often used as a technique in dental clinics to ameliorate fear, and findings that self-distraction is one of the most common child coping strategies [39] is consistent with research showing a relationship between dental anxiety and a disposition in children to monitor or attend to threat-relevant information during a dental examination [40]. However, dental coping strategies are not straightforward, and vary by age, dental anxiety and previous pain experience [41]. It would be of significant benefit to further develop and extend this work using both children and adults, as better understanding coping strategies for dentally fearful people may lead to significant improvements in both dental service utilization and oral health.

One limitation of this study was the use of the single-item DAQ to measure dental fear. Although this item has previously proved useful and is convenient for a telephone interviewing survey where brevity is an issue, there are other measures that might have been usefully employed. For example, the summary item from the Dental Fear Survey [42] has previously been employed in telephone surveys [43]. Also, the 4-item Dental Anxiety Scale [44] has been extensively used as an epidemiological measure of trait dental anxiety and has good psychometric properties and considerable normative data. It must be recognised that while single-item measures often relate well to full-scale scores and can be used to obtain a reasonably reliable estimate of global fear, they are generally poorly equipped to assess the many nuances and dimensions that characterise dental fear and for this reason psychometrically sound multi-item scales are preferred.

Apart from the roles that delaying dental visiting and subsequent invasive treatments are proposed to have on heightening or maintaining dental fear, a number of researchers have stressed the importance of escalating psychological factors in contributing to a vicious cycle of dental fear. For instance, catastrophizing ideations have been found among people with dental fear [45] and this is believed to impact on both the physical and emotional distress experienced during a dental examination [46] and on the perceived pain of treatment [47,48]. It has also been argued that a strong sense of embarrassment, especially following many years of avoidance, related to feelings of self-punishment, shame and negative self-image may be an important aspect of a vicious cycle of dental anxiety [15,18]. While this paper did not look at these various psychological factors, it is quite likely that these and other cognitive and emotional components help to facilitate the progression in the vicious cycle involving fear and dental decay.

This study found almost one in three people with high dental fear fit the profile hypothesised by a vicious cycle of dental fear, having delayed dental visiting, poorer oral health and symptomatic dental visiting patterns. The idea of a vicious cycle of dental fear can be used to describe the specific clustering of detrimental behavioural and oral health outcomes in some people, which may serve to perpetuate or even exacerbate the anxiety and fear associated with dental visiting. In future research there may be value in attempting to differentiate between people with high dental fear and those who might have potentially diagnosable dental phobia as well as look at differences in coping strategies of both high- and low-fear people and of high-fear people who fit a vicious cycle profile and high-fear people who manage to maintain regular dental visiting patterns.

## Conclusion

This study found a relatively high prevalence of 11.9 percent of people with very high dental fear in a large, representative, national sample of the Australian population. Extrapolated to the population, this equates to about two and a half million Australians suffering very high dental fear and reconfirms the scope of the problem facing dentists and policy makers in improving the generally poor oral health of, and symptom-oriented treatment received by, these people. Individuals with dental fear represent a particularly difficult population to treat and present special challenges to dental staff in terms of the management of care. While efforts are being made to reduce the incidence of dental fear among younger Australians who may be visiting the dentist for the first time, a concerted effort is also required to break what appears to be a vicious cycle of dental fear and provide assistance to those individuals with established fear-avoidance patterns.

## Competing interests

The authors declare that they have no competing interests.

## Authors' contributions

JA conceived of the paper, performed the statistical analysis and wrote the manuscript. AJS conceived of the study, participated in its design and helped to draft the manuscript. JS helped coordinate the study and provided some assistance in drafting the manuscript. All authors have read and approved the final manuscript.

## Pre-publication history

The pre-publication history for this paper can be accessed here:



## References

[B1] Smith TA, Heaton LJ (2003). Fear of dental care: are we making any progress?. J Am Dent Assoc.

[B2] Lindsay SJ, Humphris G, Barnby GJ (1987). Expectations and preferences for routine dentistry in anxious adult patients. Br Dent J.

[B3] Folayan MO, Idehen EE, Ojo OO (2004). The modulating effect of culture on the expression of dental anxiety in children: a literature review. Int J Paediatr Dent.

[B4] Armfield JM, Spencer AJ, Stewart JF (2006). Dental fear in Australia: Who's afraid of the dentist?. Aust Dent J.

[B5] Thomson WM, Stewart JF, Carter KD, Spencer AJ (1996). Dental anxiety among Australians. Int Dent J.

[B6] Schuller AA, Willumsen T, Holst D (2003). Are there differences in oral health and oral health behavior between individuals with high and low dental fear?. Community Dent Oral Epidemiol.

[B7] Taani DQ (2002). Dental attendance and anxiety among public and private school children in Jordan. Int Dent J.

[B8] Sohn W, Ismail AI (2005). Regular dental visits and dental anxiety in an adult dentate population. J Am Dent Assoc.

[B9] Nuttall NM (1984). Characteristics of dentally successful and dentally unsuccessful adults. Community Dent Oral Epidemiol.

[B10] Moore R, Birn H (1990). [Phenomenon of dental fear]. Tandlaegebladet.

[B11] Milgrom P, Weinstein P (1993). Dental fears in general practice: new guidelines for assessment and treatment. Int Dent J.

[B12] Abrahamsson KH, Berggren U, Hallberg L, Carlsson SG (2002). Dental phobic patients' view of dental anxiety and experiences in dental care: a qualitative study. Scand J Caring Sci.

[B13] Folayan MO, Idehen E (2004). Factors influencing the use of behavioral management techniques during child management by dentists. J Clin Pediatr Dent.

[B14] Bouma J, Uitenbroek D, Westert G, Schaub RM, van de Poel F (1987). Pathways to full mouth extraction. Community Dent Oral Epidemiol.

[B15] Berggren U, Meynert G (1984). Dental fear and avoidance: causes, symptoms, and consequences. J Am Dent Assoc.

[B16] Weinstein P (1990). Breaking the worldwide cycle of pain, fear and avoidance: uncovering risk factors and promoting prevention. Ann Behav Med.

[B17] Locker D (2003). Psychosocial consequences of dental fear and anxiety. Community Dent Oral Epidemiol.

[B18] Moore R, Brodsgaard I, Rosenberg N (2004). The contribution of embarrassment to phobic dental anxiety: a qualitative research study. BMC Psychiatry.

[B19] Carter KD, Stewart JF (2003). National Dental Telephone Interview Survey 2002.

[B20] Frankel MR, Frankel LR (1977). Some recent developments in sample survey design. J Market Res.

[B21] Dillman DA (1978). Mail and telephone surveys: the total design method.

[B22] Neverlien PO (1990). Assessment of a single-item dental anxiety question. Acta Odontol Scand.

[B23] Neverlien PO, Backer Johnsen T (1991). Optimism-pessimism dimension and dental anxiety in children aged 10-12 years. Community Dent Oral Epidemiol.

[B24] Locker D, Shapiro D, Liddell A (1996). Who is dentally anxious? Concordance between measures of dental anxiety. Community Dent Oral Epidemiol.

[B25] Slade GD, Spencer AJ (1994). Development and evaluation of the Oral Health Impact Profile. Community Dent Health.

[B26] Slade GD (1997). Derivation and validation of a short-form oral health impact profile. Community Dent Oral Epidemiol.

[B27] Department of Health and Aged Care and the National Key Centre for Social Applications of Geographical Information Systems (1999). Measuring remoteness: accessibility/remoteness index of Australia (ARIA). Occassional Papers Series.

[B28] Kleinknecht RA, Klepac RK, Alexander LD (1973). Origins and characteristics of fear of dentistry. J Am Dent Assoc.

[B29] Davey GC (1989). Dental phobias and anxieties: evidence for conditioning processes in the acquisition and modulation of a learned fear. Behav Res Ther.

[B30] Milgrom P, Mancl L, King B, Weinstein P (1995). Origins of childhood dental fear. Behav Res Ther.

[B31] Locker D, Shapiro D, Liddell A (1996). Negative dental experiences and their relationship to dental anxiety. Community Dent Health.

[B32] De Jongh A, van der Burg J, van Overmeir M, Aartman I, van Zuuren FJ (2002). Trauma-related sequelae in individuals with a high level of dental anxiety. Does this interfere with treatment outcome?. Behav Res Ther.

[B33] Armfield JM (2006). Cognitive vulnerability: a model of the etiology of fear. Clin Psychol Rev.

[B34] Chattopadhyay A, Kumar JV, Green EL (2003). The New York State Minority Health Survey: determinants of oral health care utilization. J Public Health Dent.

[B35] Lo GL (1993). The use of dental services by adult Singaporeans. Singapore Dent J.

[B36] Nuttall NM (1996). Initial development of a scale to measure dental indifference. Community Dent Oral Epidemiol.

[B37] Craven RC, Blinkhorn AS, Schou L (1994). A campaign encouraging dental attendance among adolescents in Scotland: the barriers to behaviour change. Community Dent Health.

[B38] American Psychiatric Association (1994). Diagnostic and statistical manual of mental disorders (4th ed.).

[B39] Versloot J, Veerkamp JS, Hoogstraten J, Martens LC (2004). Children's coping with pain during dental care. Community Dent Oral Epidemiol.

[B40] Miller SM, Roussi P, Caputo GC, Kruus L (1995). Patterns of children's coping with an aversive dental treatment. Health Psychol.

[B41] Van Meurs P, Howard KE, Versloot J, Veerkamp JS, Freeman R (2005). Child coping strategies, dental anxiety and dental treatment: the influence of age, gender and childhood caries prevalence. Eur J Paediatr Dent.

[B42] Kleinknecht EE, Bernstein DA (1978). The assessment of dental fear. Behav Res Ther.

[B43] Moore R, Birn H, Kirkegaard E, Brodsgaard I, Scheutz F (1993). Prevalence and characteristics of dental anxiety in Danish adults. Community Dent Oral Epidemiol.

[B44] Corah NL (1969). Development of a dental anxiety scale. J Dent Res.

[B45] de Jongh A, Muris P, Schoenmakers N, Ter Horst G (1995). Negative cognitions of dental phobics: reliability and validity of the Dental Cognitions Quesionnaire. Behav Res Ther.

[B46] Sullivan MJ, Neish N Catastrophic thinking and the experience of pain during dental procedures. J Indiana Dent Assoc.

[B47] Sullivan MJ, Neish NR (1998). Catastrophizing, anxiety and pain during dental hygiene treatment. Community Dent Oral Epidemiol.

[B48] Sullivan MJ, Thorn B, Rodgers W, Ward LC (2004). Path model of psychological antecedents to pain experience: experimental and clinical findings. Clin J Pain.

